# A comprehensive proteomic analysis of umbilical cord blood supports COVID-19 vaccination before pregnancy

**DOI:** 10.1038/s41392-024-02024-7

**Published:** 2024-11-20

**Authors:** Jianbin Guo, Xiaoyue Tang, Roujie Huang, Jiangfeng Liu, Lan Zhu

**Affiliations:** 1 Department of Obstetrics and Gynecology, National Clinical Research Center for Obstetric & Gynecologic Diseases, the State Key Laboratory for Complex, Severe, and Rare Diseases, Peking Union Medical College Hospital, Chinese Academy of Medical Sciences and Peking Union Medical College, Beijing, China; 2 State Key Laboratory of Common Mechanism Research for Major Diseases, Department of Biochemistry and Molecular Biology, Institute of Basic Medical Sciences Chinese Academy of Medical Sciences, School of Basic Medicine Peking Union Medical College, Beijing, China

**Keywords:** Vaccines, Infection, Infectious diseases, Vaccines, Reproductive disorders

**Dear Editor,**

Coronavirus disease 2019 (COVID-19) is a serious respiratory disease caused by severe acute respiratory syndrome coronavirus 2 (SARS-CoV-2). Since the emergence of SARS-CoV-2, extensive research has been conducted to develop safe and effective vaccines against COVID-19, including messenger RNA (mRNA), viral vector, inactivated vaccines and protein subunit vaccines.

The correlations among vaccine dose, efficacy, and vaccine-induced molecular alterations have been explored in adults. However, pregnant women—a particularly vulnerable segment of the population—may still exhibit vaccine hesitancy due to safety concerns.^1^ Some studies underscore the safety of COVID-19 vaccination during pregnancy and encourage pregnant women to actively seek vaccination.^2^ However, they focused primarily on individuals who were vaccinated during pregnancy and rarely considered the vaccination history of pregnant women. Besides, the population of previous studies was predominantly non-Asian. Given the heterogeneity in vaccine responses across populations and differences in vaccine types—that is, given that most mRNA vaccines are used abroad, while most inactivated vaccines are used in China-studies among Chinese populations are still needed to fill this gap. Therefore, it is necessary to explore whether pregnant women in China should be vaccinated based on the current background. Herein, we collected umbilical cord blood samples from 159 women who were vaccinated prior to the onset of pregnancy and 56 unvaccinated women (Fig. 1a). All of them were infected during pregnancy with different intrauterine recovery times (from infection to delivery, IRT).

**Fig. 1:**
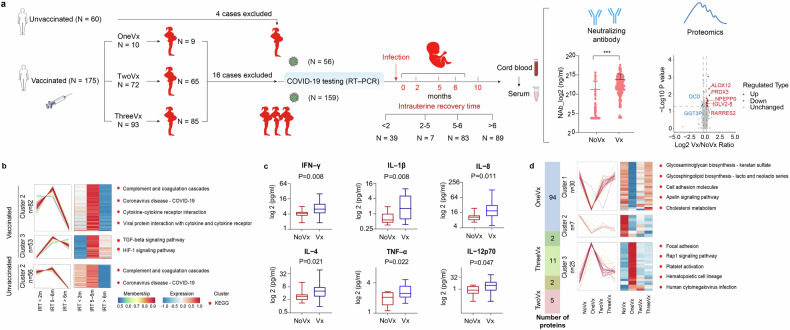
A history of maternal vaccination against SARS-CoV-2 benefits the fetus in utero. **a** Schematic view of the research cohort and the experimental procedure. Umbilical cord blood samples were collected from 175 women who were vaccinated prior to the onset of pregnancy (159 infected during pregnancy) and 60 unvaccinated women (56 infected). The number of vaccinations administered and the time from the mother’s infection to the delivery of each newborn (intrauterine recovery time, IRT) were recorded. Comparisons of the mean neutralizing antibody concentration and differentially expressed proteins were performed between the vaccinated group and the unvaccinated group among individuals infected with SARS-CoV-2 during pregnancy. **b** Clusters of protein expression across different IRT. Differently expressed proteins (ANOVA p < 0.05) were analyzed using k-means clustering. Pathway enrichment analysis of proteins in each cluster identified the top associated enriched KEGG terms showing on the right. **c** Comparison of the cytokine concentrations in umbilical cord plasma between vaccinated group and unvaccinated group. Six cytokines showed significant differences in participants with an IRT of less than 2 months. **d** The overlap of the number of differently expressed proteins between women who received different doses of vaccine (including one, two and three) and unvaccinated women, and clusters of protein expression across different doses. Differently expressed proteins (ANOVA *p* < 0.05) were analyzed using k-means clustering. Pathway enrichment analysis of proteins in each cluster identified the top associated enriched KEGG terms showing on the right

Plasma-neutralizing antibodies against SARS-CoV-2 were measured. A history of vaccination increased the concentration of neutralizing antibodies in the fetal circulation among pregnant women infected with SARS-CoV-2 during pregnancy (Fig. 1a). The proteomic approach resulted in 872 quantified proteins in the umbilical cord plasma. Some proteins were up-regulated when comparing the vaccinated group to the unvaccinated group, such as immunoglobulin lambda variable 2-8 (IGLV2-8), puromycin-sensitive aminopeptidase (NPEPPS), and retinoic acid receptor responder 2 (RARRES2) (Fig. 1a). IGLV2-8 and NPEPPS directly participate in immune responses, and RARRES2 has both pro- and anti-inflammatory properties depending on the modality of enzymatic cleavage by different classes of proteases. Trend analysis of differently expressed proteins (DEPs) was conducted based on IRT in vaccinated and nonvaccinated groups, respectively (Fig. 1b). The complement and coagulation cascades pathway, which is related to thrombosis, a sequela of COVID-19,^3^ and the coronavirus disease-COVID-19 pathway were enriched in both two groups, characterized by the activation and subsequent inhibition (Fig. 1b). Pathways associated with immunity, such as cytokine‒cytokine receptor interactions, viral protein interactions with cytokine and cytokine receptor pathways, and the transforming growth factor-beta (TGF-β) signaling pathway, were enriched in the vaccinated group. Considering the immunoregulatory effects of cytokines, twelve cytokines were assessed for validation, and 6 cytokines with significant differences in expression between the vaccinated and nonvaccinated groups were identified within 2 months of intrauterine recovery (Fig. 1c). At IRT of 5-6 months and over 6 months of intrauterine recovery, cytokine levels tended to be similar between the two groups. Taken together, these results revealed the IRT-dependent trend of protein signatures in the umbilical cord blood of vaccinated pregnant women, characterizing the process of intrauterine recovery and suggested that maternal vaccination against SARS-CoV-2 enhances immune regulation in the fetal circulation after infection.

The samples for this study were collected after a period of infection, representing the process of recovery from illness (rather than immune response in an infected state). We attempted to determine the impact of various vaccine doses during the recovery period after infection via proteomics analysis. Comparisons were performed between women who received different doses of vaccine (including one, two and three) and unvaccinated women. The results revealed that the number of differentially expressed proteins in the one-dose vaccinated group (96 DEPs) was significantly greater than that in the two-dose (15 DEPs) and three-dose (7 DEPS) vaccinated groups (Fig. 1d). To further advance our understanding of the dynamic protein-regulatory network at different doses, trend analysis revealed that the platelet activation pathway, which is related to long COVID-19^4^ was activated in the group of women who had received one dose of the vaccine (Fig. 1d). The related proteins of this pathway, such as integrin beta-3 (ITGB3), platelet glycoprotein Ib beta chain (GP1BB), and von Willebrand Factor (vWF), were highly expressed after the first injection while not differentially expressed after the second and third injections (Fig. 1d). Proteins with similar trends included FERM domain containing kindlin 3 (FERMT3, associated with integrin-regulated platelet adhesion), glycoprotein IX platelet (GP9, involved in regulating vWF-dependent platelet adhesion), triggering receptor expressed on myeloid cells like 1 (TREML1, engaged in platelet aggregation), and glycoprotein Ib platelet subunit beta (GP1BB, involved in the formation of platelet plugs by binding to vWF) (Fig. 1d). Along with the inhibition of the Rap1 pathway at 2 and 3 vaccination doses, which promotes the inflammatory response,^5^ platelet activation pathway was significantly weakened. According to the above results, in contrast to the relatively persistent immune dysregulation and platelet activation in the fetal circulation with one dose of the vaccine, two or three doses of the vaccine demonstrated protective effects on the intrauterine fetus. It can thus be speculated that full vaccination contributes to the induction of appropriate immune responses and the maintenance of immune homeostasis.

This is the first study on the impact of inactivated virus vaccines on fetal circulating immunity, which is highly important for understanding the immune status of Chinese fetuses against SARS-CoV-2 in utero. There were some limitations in this study. Firstly, blood of pregnant women was not collected and correlation between maternal and fetal circulation could not be analyzed. Secondly, almost no umbilical cord blood plasma was collected during infection; therefore, changes in fetal circulation during SARS-CoV-2 infection could not be assessed. In addition, we did not continue to test for changes in blood after birth, because the mother refused to collect blood from healthy babies.

In summary, we used very precious samples to study the impact of vaccination history before pregnancy on the intrauterine recovery of fetuses from mothers infected with SARS-CoV-2 during pregnancy in China. Considering that China implemented a stringent zero-COVID policy until December 2022, the population included in our research is relatively homogeneous. They had no history of SARS-CoV-2 infection before pregnancy, and most of them were vaccinated before pregnancy and infected during pregnancy at a similar time. It is extremely difficult to include such a group in the real world to monitor the effects of maternal vaccination and subsequent infection on fetuses. A history of vaccination before pregnancy not only increased neutralizing antibody levels in umbilical cord blood but also enhanced immune regulation. Multiple vaccine doses are recommended for achieving a more appropriate fetal circulating immunological response than one dose. More importantly, the protective effect of the SARS-CoV-2 inactivated vaccine on the fetus, to a certain extent, can be extended to other pathogen infections and vaccines and helps us understand the vaccination and immune response to infection during pregnancy.

## Supplementary information

Supplementary methods

## Data Availability

The proteomics data have been deposited to the ProteomeXchange Consortium (http://proteomecentral.proteomexchange.org) via the iProX partner repository with the dataset identifier PXD056208.
