# Supplementary material for: A comprehensive proteomic analysis of umbilical cord blood supports COVID-19 vaccination before pregnancy
Source: Signal Transduct Target Ther. 2024 Nov 20;9:315. doi: 10.1038/s41392-024-02024-7 (PMC11577047; doi:10.1038/s41392-024-02024-7)
Supplement: Supplementary file 1 — Supplementary methods [file 41392_2024_2024_MOESM1_ESM.docx]

Supplementary Materials and Methods for

A comprehensive proteomic analysis of umbilical cord blood supports COVID-19 vaccination before pregnancy

Jianbin Guo^1^†, Xiaoyue Tang^2^†, Roujie Huang^1^†, Jiangfeng Liu^2^*, Lan Zhu^1^*

Correspondence to: zhu_julie@vip.sina.com

**This file includes:**

Materials and Methods

**Materials and Methods**

**Samples Collection**

Umbilical cord blood was collected from pregnant women who gave birth in the labor room or undergone cesarean section in the operating room in Peking Union Medical College Hospital. The study was approved by the Institutional Review Board of Peking Union Medical College Hospital (No. I-23PJ901) and performed in compliance with the Declaration of Helsinki. Blood was collected in Ethylenediaminetetraacetic acid tubes (EDTA) and stored at 4℃ for less than 6 hours. Centrifuge the tubes at 3000 rpm/min for 5 min. Supernatant is preserved at -80℃.

**Cohort information**

The types of vaccines administered to participants were the Sinovac COVID-19 vaccine and the Sinopharm COVID-19 vaccine. Both vaccines have been extensively studied and approved for public use by China’s medical product regulators. All infections were confirmed by a real-time reverse transcription–polymerase chain reaction (RT‒PCR) assay to detect SARS-CoV-2 nucleic acid. The inclusion criteria were as follows: (1) age > 18 years; (2) provided the quantity of vaccination; (3) clear infection status confirmed by RT‒PCR assay; and (4) live births ≥ 28 weeks. The exclusion criteria were as follows. (1) inability to provide the quantity of vaccination; (2) pregnant women with unknown infection status: a. COVID-19-related symptoms, such as fever and cough, occurred during pregnancy, but no RT‒PCR assay was performed; b. no COVID-19-related symptoms, and no RT‒PCR assay was performed; (3) miscarriage at < 28 weeks.

We collected umbilical cord blood samples from 175 women who were vaccinated prior to the onset of pregnancy (159 infected during pregnancy) and 60 unvaccinated women (56 infected). Samples from 20 pregnant women who were not diagnosed with infection were collected, but excluded in subsequent analysis due to the inability to be determined whether they were asymptomatic carriers. Two hundred and fifteen pregnant women with confirmed infections by SARS-CoV-2 during pregnancy were included in the final analysis, and the IRTs were recorded. Among the 159 vaccinated women, 9 individuals had received one dose of the COVID-19 inactivated vaccine, 65 had received two doses of the vaccine, and 85 had received three doses of the vaccine. Because four women conceived twins, 249 plasma samples (218 infected) were collected, including 39 with an IRT of less than 2 months, 7 with an IRT between 2–5 months, 83 with an IRT between 5–6 months, 89 with an IRT greater than 6 months. Among the 159 vaccinated individuals, the median time from the last vaccine to delivery was 17.4 (6.2-29.2) months. All pregnant women had no history of SARS-CoV-2 infection before pregnancy.

**Neutralization antibody measurement and Cytokines Detection**

Neutralizing antibodies were measured with SARS-CoV-2 Neutralization Antibody ELISA Kit from Elabscience (Catalog No: E-EL-E608). EasyMagPlex Human Cytokine 12 Plex Kit from Wellgrow Technology Ltd. was used to detect 12 cytokines including IL-1β, IL-, IL-4, IL-5, IL-6, IL-8, IL-10, IL-12p70, IL-17, TNF-α, IFN-γ, IFN-α.

**Protein extraction**

Plasma proteins were extracted using Micro Sample Preparation Kit (Omicsolution). Briefly, the protein concentration was determined using BCA protein assay kit (Thermo Scientific, Branchburg, NJ, United States). A total of 30 μg of protein per sample was used and then prepared with the Micro Sample Preparation Kit according to the instructions. After proteolysis at 37°C for 2 h, the samples were desalted, dried, and dissolved in 20 μL of 0.1% formic acid (FA) for subsequent liquid chromatography-tandem mass spectrometry (LC-MS/MS) analysis.

**Direct data-dependent analysis (DIA) proteomics analysis**

UltiMateTM 3000 UHPLC (Thermo Scientific) and timsTOF Pro2(Bruker Daltonics) were used to perform the LC-MS/MS analysis. After loading all the samples onto the trap column, the eluent was further passed through a reversed-phase analytical column (75 mm × 500 mm, 2 mm, MONOTECH). To separate peptides, an 18-minute gradient was applied to a reversed-phase analytical column (75 mm × 500 mm, 2 mm, MONOTECH) at a flow rate of 500nL/min for peptide separation and 1500 nl/min for system wash. Mobile phase B consisted of 80% acetonitrile in water, whereas mobile phase A involved 0.1% formic acid in water. Mass-spectrometric data were acquired using the parallel accumulation serial fragmentation (PASEF) acquisition method in DIA mode with a 22-windows method using 40 Da windows covering the mobility ranges over 0.71- 1.26 1/K0 [V·s/cm^2^]. The m/z range default was 349.2-1229.2 with an ion mobility range of 0.71- 1.26 1/K0 [V·s/cm^2^], which corresponded to an estimated cycle time of 1.48 sec. DIA-PASEF windows and collision energy were also left to default with base of 0.601/K0 [V·s/cm^2^] set at 20 eV and 1.601/K0 [V·s/cm^2^] set at 59 eV. TIMS and mass calibration were performed linearly using three calibrant ions at 622, 922, and 1222 m/z (Agilent) at different ranges to match Δ1/K0 of each respective set of ranges.

The raw data from DIA proteomics analysis were searched using Spectronaut Pulsar X (Biognosys, AG, Schlieren, Switzerland) software with default settings. An optimal XIC extraction window was identified based on the source-specific indexed retention time (iRT) calibration strategy. The mass tolerance strategy was set to dynamic based on extensive mass calibration. The cross-run normalization was set to local normalization based on local regression. The sum peak areas of the respective fragment ions in MS2 were used to quantify peptide intensities.

**Bioinformatics and statistical analyses**

Quantitative data were further analyzed and visualized using Perseus (version 1.6.14.0). Data were filtered to remove potential contaminants, reverse hits, and protein groups “only identified by site”. Protein intensities were Log_2_ transformed. Statistical comparisons between two groups were performed with Student’s t-test (two-tailed) and the cutoff of adjusted p-value < 0.05 with Benjamini & Hochberg correction and fold change>1.2 or <0.833 were used to select differential proteins. The enrichment analysis was performed by the hypergeometric test based on the KEGG (Kyoto Encyclopedia of Genes and Genomes) annotation. Statistical comparisons among three groups were performed with analysis of variance (ANOVA). Proteins with a cutoff of p<0.05 (FDR adjusted) were then subjected for trend analysis. The discrete clusters of proteins with similar expression changes were identified on fuzzy c-means algorithm implemented with Mfuzz (v. 2.48.0) through R package (v. 3.6). A non-negative matrix factorization (NMF) clustering algorithm was used to cluster all of the samples. And related data were visualized was performed using GraphPad Prism Software Version 8.0.2 (San Diego, CA, USA). For comparisons of continuous variables across multiple groups, one-way analysis of variance (ANOVA) was performed.
